# Postpartum Care Service Experience Model: A Case of Postpartum Home Visiting Services

**DOI:** 10.3390/healthcare6030091

**Published:** 2018-08-01

**Authors:** Pei-Ping Chang

**Affiliations:** Department of Early Childhood Care and Education, Tzu Hui Institute of Technology, Pingtung 926, Taiwan; paiping@gmail.com; Tel.: +886-8-864-7367 (ext. 291)

**Keywords:** service experience model, postpartum care, satisfaction

## Abstract

The choice of postpartum care methods has expanded with changes in population structure and social forms. In addition to the traditional provision of postpartum assistance by senior members of the parents’ family, recent years have also seen a rapid growth in care centers and postpartum home visiting services. Postpartum home visiting services combine the features and services of senior family members’ assistance and postpartum care centers. Therefore, special attention has been drawn to the postpartum care service experience. This study conducted a survey among mothers participating in the Postpartum Home Visiting Service Project of the Social Affairs Bureau of Kaohsiung City Government, Taiwan. The effective sample included 43 participants. Partial least squares (PLS) analysis was performed. The results indicated that the service experience model could comprehensively reflect the relation between consumer experience, experiential value, and experience satisfaction. With regard to practical implications, the results provide postpartum home visiting service providers with a better understanding of consumers’ feelings and, thus, put them in a better position to improve experience through appropriate service methods.

## 1. Introduction

Traditionally, postpartum care behaviors have been widely maintained in Chinese society and are considered important from the perspective of maintaining customs and one’s health. Therefore, postpartum care has not been affected or destabilized by time. Postpartum care requirements have received more attention due to changes in family structure and a low birth rate, giving rise to various new forms of postpartum care services and providing women with an expanded choice of postpartum care methods. Three types of postpartum care can be distinguished in modern Chinese society: (1) assistance from senior family members; (2) postpartum care centers; and (3) postpartum home visiting service [1,2]. Postpartum home visiting service allows for the kind of family support made possible through senior family members’ assistance, as well as the provision of newborn care, attendant support to mothers, and one-month food preparation by professional personnel from postpartum care centers. As such, a mother can enjoy a sufficient level of rest.

**Figure 1:**
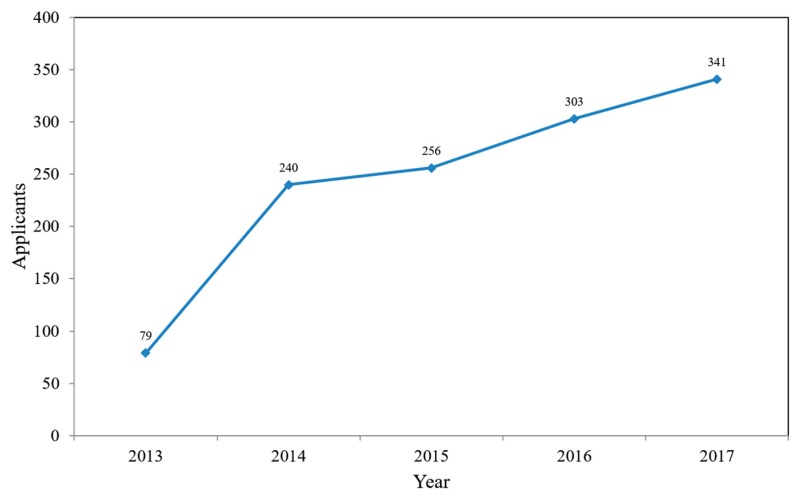
Growth of the number of postpartum home visiting service applicants from 2013 to 2017.

The Postpartum Home Visiting Service Project was established by the Social Affairs Bureau of Kaohsiung City Government, Taiwan on 1 April 2013. The number of mothers who applied for the postpartum home visiting service increased from 79 in 2013 to 240 in 2014, 256 in 2015, 303 in 2016, and 341 in 2017 (Figure 1), indicating a growing attention to the postpartum home visiting service. Necessity of discussing the postpartum home visiting service was the first motivation for this study.

According to Pine and Gilmore [3], the economy has stepped into the era of the experience economy. Schmitt [4] proposed the concept of experiential marketing consisting of five major strategic experiential modules and suggested that experiential marketing focuses on customer experience and pays attention to the consumer context. Customers are stimulated by rational and emotional drivers. Diverse methods must be used to observe consumers’ experiences. Brakus [5] discussed different cognitive foundations of the five experiential modules proposed by Schmitt. Recent studies have focused on consumer experiences in their research on medical services [6,7], blog browsing [8], clothing consumption [9], tourism industry [10], and food brands [11].

The postpartum home visiting service enables mothers to receive postpartum care from personnel (maternity matron) with professional knowledge and extensive experience, while allowing them to remain in a familiar environment. The importance of exploring mothers’ experience of the postpartum home visiting service was the second motivation for this study. Sinha and DeSarbo [12] maintained that value is a subjective idea resulting from experience or its interaction effect and can be largely influenced by the context. Holbrook [13] defined customer value as an interactive and relative experience of preference, emphasizing that value is preference that is affected by an individual’s interests. Value involves an interaction with an object that can refer to a product, service, or person. Value is relative and is determined by an individual’s subjective evaluation. Value is related to experience, meaning that in true consumption, value is formed not by the product itself but by the use of the product or service. Overby [14] indicated that the value concept must include culture, product, person, and context.

Past studies on experiential value mainly discussed medical services, blog browsing, clothing consumption contexts, tourism, and food brands. No research has been done with regard to experiential value and satisfaction related to the postpartum home visiting service. The postpartum home visiting service experience is based on consumer experience. Thus, the experiential value and satisfaction level formed by consumers must be explored from the perspective of their experience, which was the third motivation for this study.

In sum, the main objective of this study was to explore the service experience model of the postpartum home visiting service. In addition to the analysis of the relation between consumer experience, experiential value, and experience satisfaction, this study aimed to develop the service experience model of the postpartum home visiting service to provide a reference for decision-making in the postpartum care industry that caters to Chinese communities.

## 2. Literature Review

### 2.1. Studies on Postpartum Care

According to the World Health Organization (WHO) [15], the terms “postpartum period” and “postnatal period” are often used interchangeably but sometimes separately, where “postpartum” refers to issues pertaining to the mother and “postnatal” refers to those concerning the baby. The period begins immediately after the birth of the baby and extends up to six weeks (42 days) after birth.

Postpartum care is of high importance in Chinese society and has been studied from cultural, medical, dietary, and social perspectives. Six main research topics can be distinguished, which are compliance with postpartum care taboos in the postpartum period, pressure and support in the postpartum care period, postpartum care and physical concepts, the meaning of postpartum care for women, quality of life in postpartum care, and the mother-daughter relationship in postpartum care [16].

With the rise of gender equality consciousness and health consciousness, many women have realized the importance of the expression of feelings, development of self-identity, and receipt of others’ support and affirmation during the postpartum period. Families opting for postpartum nursing care pay attention to the aspects of “public opinions and service” and “facilities and prices”; requirements regarding the aspect of “professional care services” have also increased [17]. Su et al. [18] found that the main reasons for mothers’ preference of institutional care included professional care, lack of people who could assist in postpartum care at home, and recommendation of colleagues and friends; other factors included better quality of care, nursery facilities, and living environment. Hori et al. [19] found that the functionality of a postpartum care center affected consumers’ intention to use it. Furthermore, services provided by a postpartum care center played a mediating role between its functionality and buying intention. Consumers were found to attach importance to 24-h customer-oriented medical team service, postnatal diet, and postpartum physical and psychological rehabilitation provided in the postpartum care center. Many women also expressed high expectations with respect to room cleanliness and good Internet connection.

Tsai et al. [20] conducted research on postpartum confinement and found that the main concerns of women included the price, location convenience, and feeling of safety provided by service personnel. Customer loyalty was found to be significantly correlated with postpartum product and service buying intention (service use, sleep quality, and care experience) and self-planning (mood, work attitude, professional knowledge, work field, and company support). Moreover, self-planning abilities were significantly affected by some of the characteristics of first-line service personnel, including sense of humor, years of experience, and ability to respond.

Based on the studies reviewed above, with regard to postpartum care, women pay attention to professional care received by them and the newborn, postnatal diet, physical and psychological rehabilitation, and characteristics of service personnel. Besides, according to Taiwanese custom, the duration of postpartum care usually lasts for 30 days. Therefore, this present study studied postpartum home visiting services by providing a 30-day professional home visiting service to women. A questionnaire was designed based on the factors described above and a service experience model was developed for the postpartum care service. The relation between service experience, experiential value, and experience satisfaction was explored.

### 2.2. Consumer Experience

Hirschman and Holbrook [21] indicated the importance of a happy experience of consumer behaviors and proposed three components of consumer experience, which were fantasies, feelings, and fun (3F). Holbrook and Hirschman [13] further developed this concept in their research on happy consumers and analyzed consumer behaviors from the perspective of the 3F. Many past studies on marketing and consumer behavior employed the concept of “information processing”, while giving little attention to leisure activities, aesthetics, and emotional response. Therefore, the concept of “consumer experience” was proposed.

Consumer experience is formed through direct observation or participation by the consumer [3]. Rifkin [22] suggested that consumer experience is the experience of a consumer that tends toward leaving a deep impression and being difficult to forget. The “experiential marketing” concept proposed by Schmitt [4] referred to marketing based on shaping consumers’ experiences. As experience is normally not spontaneous but triggered and there are no two identical experiences, experiential marketing may become mainstream in modern marketing. Carù and Cova [23] defined the range of experiential marketing as “unusual consumer experience” by distinguishing different experience degrees (usual or unusual) and consumer experience.

According to Schmitt [4], the core of experiential marketing is in the creation of different experience formats for customers with the aim of creating a complete customer experience. In the complete customer experience model, emotional experience involves an understanding of customer moods and quick response to them. Cognitive experience refers to customers’ cognitive and problem-solving experience formed using creative methods. As a result, customers are induced to think more about a company or brand in a creative manner, which promotes their re-evaluation of the company or product; customer participation leads to a paradigm shift in the company.

Multiple forms of postpartum care have emerged. In the market-oriented competitive environment, the postpartum home visiting service, as a service provided by a professional visiting a mother’s home, requires attention to professionalism and human-centered interaction. Comprehensive perception is created in the process of service provision. Thus, this study used the perspective of consumer experience to examine the service experience model provided to the consumers of the postpartum home visiting service.

### 2.3. Experiential Value

Park and Young [24] distinguished three categories of consumer needs: (1) functional needs: consumers hope to solve external needs to satisfy their basic needs or complete functional tasks (e.g., solve current problems, prevent potential future problems, resolve conflicts, etc.); (2) experiential needs: needs related to sensory pleasure, diversity, and cognitive stimulation, where consumption experience results are of high importance; and (3) symbolic needs: needs related to social relations and self-actualization. Consumers seek self-affirmation or others’ approval through consumption, which includes the improvement of one’s self-image, manifestation of one’s role and position, enhancement of group relations, and establishment of one’s self-identity. The three types of needs must be connected in brand concept management in order to strengthen the market performance of the brand. Sweeney and Soutar [25] distinguished the following types of customer values: (1) functional value: effectiveness originating from two aspects, which are the reduction of perceived short-term and long-term costs and the perceived value and benefits of the product; (2) emotional value: the effect originating from the emotions and feelings formed by the product; and (3) social value: the effect originating from improvement of the social self-concept. Drawing from the literature reviewed above, this study divided experiential values into affective value, functional value, and symbolic value. The following hypotheses were proposed with regard to the relation between consumer experience and experiential value.

Affective value is formed by the feelings and emotions produced by a product or service [25]. It includes consumer emotions, sensory experience, and generated feelings of joy, illusion, and pleasure [21]. Consumers attach importance to the feeling of safety provided by sensory stimulation, emotions, and information abundance, as well as the product’s special meaning. Based on this, the following hypotheses can be proposed:

**H1.**  *Emotional experience has a significant positive effect on affective value cognition.*

**H2.**  *Cognitive experience has a significant positive effect on affective value cognition.*

Functional value is related to the satisfaction of consumers’ functional needs by helping them to solve a problem, gain a function, or complete a task using a product’s functional characteristics [26]. Attitudes are cognitive results originating from the rational aspect. Therefore, the problem-solving process considered in the emotional and cognitive experience can generate functional value. Based on this, the following hypotheses can be proposed:

**H3.**  *Emotional experience has a significant positive effect on functional value.*

**H4.**  *Cognitive experience has a significant positive effect on functional value.*

Symbolic value can improve a consumer’s self-image, role and status, group affiliation, and self-differentiation attitude [24]. Consumers attach importance to interests, lifestyle, and the sense of identity developed through interaction with others. Therefore, they may be influenced by emotional experience and cognitive experience. Based on this, the following hypotheses can be proposed:

**H5.**  *Emotional experience has a significant positive effect on symbolic value.*

**H6.**  *Cognitive experience has a significant positive effect on symbolic value.*

### 2.4. Experience Satisfaction

Satisfaction is a link that is highly valued in the highly competitive service market. The satisfaction of customers with the postpartum home visiting service can help increase competitiveness. The consideration of target users’ role and status can increase their satisfaction.

Cardoza [27] was the first to propose the concept of customer satisfaction, suggesting that higher customer satisfaction can increase repurchase behaviors. According to Yu et al. [28], customer satisfaction has a major influence on corporate performance. Customer satisfaction with a product or service can be evaluated using appropriate tools, after which a service or product that better matches a customer’s expectations can be provided.

Oliver [29] considered satisfaction as the leading attitude-related variable. Consumers form their first attitudes based on expectations and adjust them depending on their satisfaction with the consumption experience as a result of an emotional response to a specific transaction. Woodruff et al. [30] suggested that customer satisfaction is an immediate emotional response to the value gained from using a product. Fornell [31] defined satisfaction as an overall perception that can be directly evaluated. Consumers compare a product or service against their criteria, which leads to differences in their perception of the product or service.

Parker and Mathews [32] distinguished two aspects in customer satisfaction: (1) result of a consumption activity or experience and (2) process. Kristensen et al. [33] suggested that customer satisfaction is a customer’s assessment of product purchase and consumption value experience, which is based on a comparison of the desired and the acquired. According to the study, satisfaction is a customer’s overall judgment of consumption results and can be measured using comprehensive methods. In sum, the value generated by a service experience can affect customer satisfaction. Based on this, the following hypotheses can be proposed:

**H7.**  *Affective value has a significant positive effect on experience satisfaction.*

**H8.**  *Functional value has a significant positive effect on experience satisfaction.*

**H9.**  *Symbolic value has a significant positive effect on experience satisfaction.*

## 3. Methods

### 3.1. Research Framework

**Figure 2:**
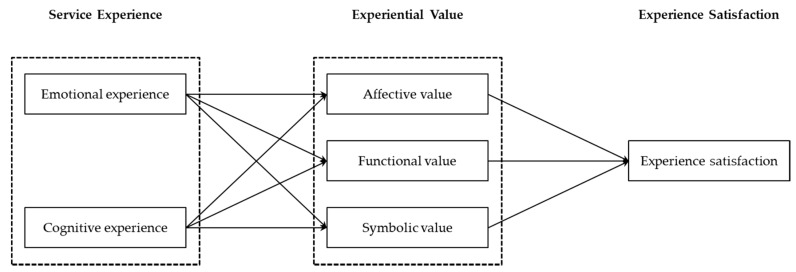
Postpartum home visiting service experience model.

The service experience model of the postpartum home visiting service established in this study is illustrated in Figure 2. The main variables in this study included service experience, experiential value, and experience satisfaction.

### 3.2. Operational Definitions of Research Variables

Operational definitions for each research aspect were derived from discussions and analyses in related studies and are provided below.

#### 3.2.1. Service Experience

This study divided the postpartum home visiting service experience into the emotional experience and cognitive experience based on the five strategic experience modules proposed by Schmitt [4]. The related references and operational definitions are shown in Table 1.

#### 3.2.2. Experiential Value

Experiential value was mainly defined based on functional, experiential, and symbolic needs proposed by Park and Young [24] and functional, affective, and social values proposed by Sweeney and Soutar [25]. Three aspects of experiential value were distinguished based on these studies and they comprise the affective value, functional value, and symbolic value. The following are their operational definitions. Affective value: feelings and emotions generated in the process of postpartum home visiting service; target service users attach importance to sensory stimulation and emotions. Functional needs: consumers hope to solve external needs to satisfy their basic needs or complete functional tasks. Symbolic value: value originating from the improvement of the social self-concept; consumers attach importance to interests, lifestyle, and the sense of identity developed through interaction with others.

#### 3.2.3. Experience Satisfaction

The definition of experience satisfaction was adopted from Kristensen et al. [33], who suggested that customer satisfaction is a customer’s assessment of product purchase and consumption value experience, which is based on a comparison of the desired and the acquired. According to the study, satisfaction is a customer’s overall judgment of consumption results and can be measured using comprehensive methods. The postpartum home visiting service is provided to mothers and their newborn children by special service personnel. Thus, these mothers’ experience satisfaction has a major influence on the operation of the postpartum home visiting service platform. Drawing from past literature, this study defined experience satisfaction as customers’ emotional response and feelings regarding the overall process of the home visiting service provided by postpartum care personnel.

### 3.3. Research Tools

#### 3.3.1. Service Experience

In this study, two experience modules consisting of five items were distinguished based on women’s considerations with regard to postpartum care and issues produced by content and interactive context of postpartum home visiting service. The items were assessed using a four-point scale. Participants gave scores between 1 (“Not satisfied”) and 4 (“Very satisfied”) based on the actual situation and degree of compliance.

#### 3.3.2. Experiential Value

Experiential value in this study was measured using a revised version of the customer value scale developed by Sweeney and Soutar [25]. Experiential value was divided into three types, namely, affective value, functional value, and symbolic value. Nine items were used to assess participants’ actual feelings and emotions. The scoring method included scores between 1 (indicating “Not satisfied”) and 4 indicating (“Very satisfied”).

#### 3.3.3. Experience Satisfaction

Experience satisfaction in this study was measured with reference to Kristensen et al. [33], who suggested that satisfaction is a customer’s overall judgment of consumption results and can be measured using comprehensive methods. One item was used to assess the overall degree of participants’ satisfaction with service. The scoring method included scores between 1 (indicating “Not satisfied”) and 4 (indicating “Very satisfied”).

### 3.4. Participants

The participants in this study were 60 women who received the postpartum home visiting service; 43 participants completed the entire research process. Prior to its implementation, the study’s compliance with research ethics was reviewed by the members of the Academic Ethics Committee of Tzu Hui Institute of Technology, and the study was evaluated to conform to the scopes of “exempt review”, announced by Ministry of Health and Welfare, Taiwan.

### 3.5. Research Process

The participants received the postpartum home visiting service for eight hours each day. The service included mother and newborn care, food preparation, and simple chores. Questionnaires were administered on the last day of the postpartum home visiting service period. The participants returned the completed questionnaires to the postpartum home visiting service platform via mail or fax. In total, 60 questionnaires were administered and 43 responses were collected, yielding the effective response rate of 71.6%.

## 4. Results

The analysis results indicated that most participants in this study (32 women) were 31–40 years old, which accounted for 74.4% of the sample. The majority of the participants (30 women; 69.89%) had a professional college/university education. With regard to their professions, the majority of the participants (11 women; 25.6%) were working in the service industry. In the majority of cases (12 women; 27.9%), the average monthly family income was NT$30,000–50,000.

In this study, model verification was conducted using partial least squares (PLS). PLS is a structural equation modeling (SEM) analysis technique that, under the least squares assumption, uses a series of independent regressions to minimize the linear structural relationship and externally measured residual variance matrix. The PLS method does not have a pre-set data distribution, which makes it unnecessary to test the data for compliance with the normality and does not require a large sample size. The issue of collinearity caused by multiple variables can be solved through simultaneous analysis of the advantages of a complex prediction model and examination of the measurement and structural models.

The model in this study involved formative constructs and the sample size was 43 participants. Therefore, the PLS method was more applicable to this study in comparison to other SEM analysis methods. This study used SmartPLS developed by Ringle et al. [37] as the analysis tool. PLS analysis and estimation steps were divided into two phases. The first phase involved a reliability and validity analysis of the measurement model. The second phase involved the evaluation and testing of the path coefficients and explanatory power of the structural model.

### 4.1. Measurement Model Reliability and Validity Analysis

In the first phase after questionnaire responses were collected, item reliability and validity were analyzed for each construct in order to verify item reliability and select the most effective factor structure. With regard to the reliability of individual items, the loading of each item was greater than 0.7. Loadings ranging between 0.63 and 0.71 indicate good reliability [38]. The results are shown in Table 2.

According to the descriptive statistics and reliability of each construct shown in Table 2, the composite reliability (CR) values of all constructs ranged between 0.82–0.94, exceeding the threshold value of 0.7 [39] and, thus, indicating internal consistency of all constructs in this study. With regard to convergent validity, as seen from Table 2, the average variance extracted (AVE) in this study ranged between 0.65–0.88, exceeding the threshold value of 0.5 and, thus, indicating convergent validity of all constructs in this study. As shown in Table 3, the mean square root of average variance extracted of all constructs was larger than the correlation coefficient, which indicated discriminant validity of all constructs in this study. With regard to content validity, this study adopted the definition of satisfaction as customers’ overall judgment of consumption results, which was proposed by Kristensen et al. [33]. The core model in this study was measurement of experience satisfaction constructs using comprehensive methods. Affective experiential factors were also incorporated into the research framework. The questionnaire’s items were developed with reference to related studies and scales and their content appropriateness was confirmed by experts. Hence, it was estimated that the questionnaire had good content validity.

### 4.2. Hypothesis Tests

**Figure 3:**
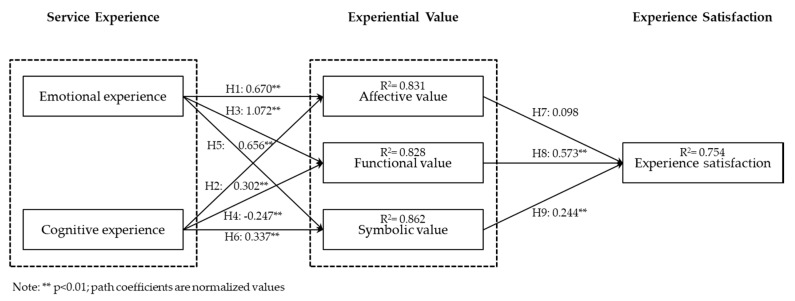
Path analysis of the structural model.

The hypotheses in this study were tested using the PLS structural model (alpha level is 0.99). The structural model was mainly used to estimate path coefficients and R^2^ values (Figure 3). Path coefficients indicate the intensity and direction of the relations between research variables and give an insight into the cause-effect relations between observable and latent variables. R^2^ refers to the proportion of variance in the dependent variable that can be explained by the explanatory variables, and indicates the predictive power of the research model. The PLS analysis results indicated that the affective value did not significantly influence customer satisfaction (β = 0.098, *p* > 0.01). As the postpartum home visiting service was provided for a short term, mother-personnel emotions were not accumulated for a long time and, therefore, affective value did not influence the overall satisfaction with service. Thus, hypothesis H7 did not receive significant support. Functional value was found to have a positive effect on customer satisfaction (β = 0.573, *p* < 0.01). The postpartum care personnel provided mothers with assistance in food preparation, house chores, newborn care, and professional child observation, immediately solving mothers’ needs. This provided mothers with a sufficient level of rest and the feeling of comfort, which directly affected their overall satisfaction with the service. Thus, hypothesis H8 received significant support. Symbolic value was found to have a positive effect on customer satisfaction (β = 0.244, *p* < 0.01). The professionalism demonstrated by the personnel during the provision of the postpartum home visiting service made women feel safe when receiving their assistance. Moreover, family members’ approval of the postpartum care personnel made women feel that their decision to receive the postpartum home visiting service was right, which affected their overall satisfaction. Thus, hypothesis H9 received significant support.

Affective value was found to be significantly affected by emotional experience (β = 0.669, *p* < 0.01) and cognitive experience (β = 0.304, *p* < 0.01), thus supporting hypotheses H1 and H2. Active and positive attitudes exhibited by the postpartum care personnel and provision of meticulous attendant care gave women the feelings of comfort and care. Women’s emotional value was met in the case of higher cognition of the postpartum care service items, satisfaction with the personnel’s service, and physical and mental pleasure.

Functional value was found to be significantly affected by emotional experience (β = 1.069, *p* < 0.01) and cognitive experience (β = −0.242, *p* < 0.01), thus supporting hypothesis H3. Due to active and positive attitudes exhibited by the postpartum care personnel and satisfaction with attendant care, women approved of the personnel’s food preparation, assistance in chores, newborn care, and professional observation. Hypothesis H4 received significant negative support. When women were well-informed about and familiar with the postpartum home visiting service, they did not require postpartum care personnel’s food preparation service, assistance in chores, newborn care, and professional observation and could perform these tasks themselves.

Symbolic value was found to be significantly affected by emotional experience (β = 0.656, *p* < 0.01) and cognitive experience (β = 0.338, *p* < 0.01), supporting hypotheses H5 and H6. In the case of active and positive attitudes exhibited by the postpartum care personnel, satisfaction with attendant care, and women’s awareness of and familiarity with postpartum care tasks, women were better able to recognize the personnel’s professionalism and their family members had positive attitudes toward postpartum care personnel.

Total variances (R^2^ values) explained by affective value, functional value, symbolic value, and satisfaction were 0.831, 0.828, 0.862, and 0.754, respectively. Thus, the model had a good explanatory power.

The analysis results indicated that the integration of experiential value factors into the service experience model improved the explanatory power of the overall model and played an important role in improving the explanatory power of the postpartum home visiting service experience value and experience satisfaction. These results can provide postpartum care platforms with a better understanding of the relation between service experience, experiential value, and experience satisfaction. Furthermore, postpartum care platforms can enhance consumers’ experiential value and satisfaction with the postpartum home visiting service by stimulating their experience and improving the personnel professionalism and quality of the postpartum home visiting service. This can increase consumers’ loyalty and willingness to recommend the postpartum home visiting service to family and friends.

## 5. Conclusions and Suggestions

The demand for the postpartum home visiting service has been increasing. This study explored the experiential value and experience satisfaction of women who had experience of receiving a professional service in a familiar environment. Participants in this study were 60 women who received the postpartum home visiting service; 43 participants completed the entire research process. The participants received the postpartum home visiting service for eight hours each day. The service included mother and newborn care, food preparation, and simple chores. Questionnaires were administered on the last day of postpartum home visiting service provision. Participants returned the completed questionnaires to the postpartum home visiting service platform via mail or fax.

A questionnaire survey was conducted among the service participants. The results showed that affective value did not influence overall satisfaction with service, meaning that women’s emotions from interaction with postpartum care personnel did not increase their overall satisfaction with service. Postpartum home visiting service was provided for a short term, whereas establishment of mother-personnel emotions required time. As an important future task, educational training can be introduced to enhance personnel’s communication and interaction skills and, thus, increase service satisfaction.

The postpartum care personnel provided mothers with assistance in food preparation, house chores, newborn care, and professional child observation, immediately solving mothers’ needs. This provided mothers with sufficient rest and the feeling of comfort, which directly affected their overall satisfaction with the service. These results show that diverse services should be included in postpartum care to provide consumers with customized and diversified choices, which is an important task for service management in future.

The professionalism demonstrated by the personnel during the provision of postpartum home visiting service made women feel safe when receiving their assistance. Moreover, family members’ approval of the postpartum care personnel made women feel that their decision to receive postpartum home visiting service was right, which affected their overall satisfaction. Thus, professionalism of postpartum care personnel is an important factor considered by women in their choice of the postpartum care institution. Postpartum care institutions must carefully examine the professionalism of personnel in order to increase their competitive advantage.

In the postnatal period, women were likely to develop psychological depression [40]. Therefore, active and positive attitudes exhibited by the postpartum care personnel and provision of meticulous attendant care gave women the feelings of comfort and care. Women’s emotional value was met in the case of the higher cognition of the postpartum care service items, satisfaction with the personnel’s service, and physical and mental pleasure.

Due to the active and positive attitudes exhibited by the postpartum care personnel and the satisfaction with attendant care, the women approved of the personnel’s food preparation, assistance in chores, newborn care, and professional observation. Moreover, when women were well-informed about and familiar with postpartum home visiting services, they did not require postpartum care personnel’s food preparation service, assistance in chores, newborn care, and professional observation and were able to perform these tasks themselves.

Finally, in the case of active and positive attitudes exhibited by the postpartum care personnel, satisfaction with attendant care, and women’s awareness of and familiarity with postpartum care tasks, women were better able to recognize the personnel’s professionalism and their family members had positive attitudes toward postpartum care personnel. The results of this study can help postpartum care institutions improve the content and quality of the home visiting service. Furthermore, professional competence and attitudes of personnel must be considered in order to achieve high satisfaction and approval of service.

## Figures and Tables

**Table 1:** Operational definitions of service experience variables.

Variables	Operational Definition	References
Emotional experience	Internal feelings and emotions of target service users, which are affected by the service context and postpartum service personnel.	Breckler and Wiggins [34]; Schmitt [4]; Schultz [35].
Cognitive experience	Target service users require sufficient amount of information to rationally evaluate service benefits.	Petty and Cacioppo [36]; Schmitt [4].

**Table 2:** Reliability and validity analysis of the postpartum service experience model.

Research Variable	Observed Variable	Mean	Std.	Factor Loading	Composite Reliability	AVE	Cronbach Alpha
Emotional experience	Your satisfaction with attendant care	3.51	0.63	0.82	0.90	0.76	0.84
	The personnel can provide active and positive interaction	3.67	0.61	0.90			
	The personnel is patient, considerate, and careful in her/his work	3.65	0.57	0.89			
Cognitive experience	Your satisfaction with the personnel’s daily record content	3.44	0.59	0.91	0.82	0.70	0.58
	Your satisfaction with the content of the postpartum care contract	3.23	0.65	0.75			
Affective value	If you have a question, the personnel can immediately give a clear reply	3.60	0.54	0.86	0.94	0.83	0.90
	Your satisfaction with the personnel’s attitude toward postpartum care	3.60	0.58	0.96			
	The personnel is responsible in each task	3.47	0.63	0.91			
Functional value	I like the food provided by the personnel	3.40	0.66	0.86	0.88	0.65	0.82
	The personnel is good at house chores	3.19	0.66	0.82			
	Newborn care provided by the personnel is professional	3.63	0.58	0.79			
	The personnel’s observation of child behavior and development is professional	3.72	0.50	0.75			
Symbolic value	Family members’ attitudes toward the personnel’s assistance at home	3.58	0.63	0.93	0.94	0.88	0.86
	Overall experience of postpartum care provided by the personnel	3.53	0.63	0.95			
Satisfaction	Satisfaction with the overall service provided by the personnel	3.47	0.63	1.00	1.00	1.00	1.00

**Table 3:** Construct correlation coefficient matrix.

	Functional Value	Cognitive Experience	Affective Value	Emotional Experience	Symbolic Value	Experience Satisfaction
**Functional Value**	0.88					
**Cognitive Experience**	0.53	0.82				
**Affective Value**	0.78	0.78	0.94			
**Emotional Experience**	0.89	0.72	0.89	0.90		
**Symbolic Value**	0.80	0.81	0.91	0.90	0.94	
**Experience Satisfaction**	0.85	0.62	0.77	0.82	0.79	1

Note: Values in the diagonal line are mean square roots of average variance extracted; values in other cells are correlation coefficients. Data source: compiled by the author.
